# Zinc Binding Directly Regulates Tau Toxicity Independent of Tau Hyperphosphorylation

**DOI:** 10.1016/j.celrep.2014.06.047

**Published:** 2014-07-24

**Authors:** Yunpeng Huang, Zhihao Wu, Yu Cao, Minglin Lang, Bingwei Lu, Bing Zhou

**Affiliations:** 1 State Key Laboratory of Biomembrane and Membrane Biotechnology, School of Life Sciences, Tsinghua University, Beijing 100084, China; 2 Department of Pathology, Stanford University School of Medicine, Stanford, CA 94305, USA

## Abstract

Tau hyperphosphorylation is thought to underlie tauopathy. Working in a *Drosophila* tauopathy model expressing a human Tau mutant (hTauR406W, or Tau*), we show that zinc contributes to the development of Tau toxicity through two independent actions: by increasing Tau phosphorylation and, more significantly, by directly binding to Tau. Elimination of zinc binding through amino acid substitution of Cys residues has a minimal effect on phosphorylation levels yet essentially eliminates Tau toxicity. The toxicity of the zinc-binding-deficient mutant Tau* (Tau*C2A) and overexpression of native *Drosophila* Tau, also lacking the corresponding zinc-binding Cys residues, are largely impervious to zinc concentration. Importantly, restoration of zinc-binding ability to Tau* by introduction of a zinc-binding residue (His) into the original Cys positions restores zinc-responsive toxicities in proportion to zinc-binding affinities. These results indicate zinc binding is a substantial contributor to tauopathy and have implications for therapy development.

## INTRODUCTION

Tau protein aggregation is found in several types of neurodegenerative diseases collectively termed “tauopathy,” which includes Alzheimer’s disease (AD), frontotemporal lobar degeneration chromosome 17, Pick’s disease, corticobasal degeneration, and the progressive supranuclear palsy (Brunden et al., 2009; Iqbal et al., 2005). AD, the most common and severe form of dementia, is alone an enormous burden to our everaging society.

Tau is a microtubule-associated protein that can bind to microtubules and regulate their dynamics. Under normal physiological conditions, Tau exhibits low levels of phosphorylation (Buée et al., 2000); however, it is hyperphosphorylated in several disease states. The abnormally hyperphosphorylated state of Tau protein can cause it to dissociate from the microtubule and aggregate into paired helical filaments (PHFs) (Kuret et al., 2005; Mazanetz and Fischer, 2007), leading to multiple downstream events and culminating in neuronal cell death (von Bergen et al., 2005). Because a detailed mechanism for Tau toxicity remains elusive, designing effective therapies for tauopathies continues to be a challenge.

Transition metals, such as copper (Cu), iron (Fe), and zinc (Zn), are indispensable for numerous fundamental biological processes but are toxic when homeostasis is disrupted (Nelson, 1999). Increasing evidence indicates that they may also be involved in human neurodegenerative diseases (Bush, 2003; Lovell et al., 1998). It has been known for decades that abnormally aggregated Tau proteins (PHFs) or so-called neurofibrillary tangles codeposit with several transition metals (Good et al., 1992), and compromised metal homeostasis has been demonstrated to be closely linked with the pathogenesis of AD and tauopathy in vivo (Atwood et al., 2000; Bush, 2003; Lang et al., 2012; Lovell et al., 1998). It was proposed that these metals, in particular zinc, could induce Tau hyperphosphorylation (Egaña et al., 2003; Kim et al., 2011; Sun et al., 2012) by activating kinases such as Raf/mitogen-activated protein kinase kinase and inhibiting phosphatases like PP2A (Kim et al., 2011; Sun et al., 2012; Xiong et al., 2013). Additionally, zinc has been reported to interact with Tau directly in vitro (Mo et al., 2009), although the in vivo significance of this direct interaction is not known. Encouraging results from phase II clinical trials showed that clioquinol (CQ), an old antibiotic that can act as a metal-chelating agent, slowed AD development (Ritchie et al., 2003), and recently, an improved derivative of CQ, PBT2, offered even more promising results (Faux et al., 2010; Lannfelt et al., 2008).

In a genetic screen to identify metal homeostasis genes that might be involved in tauopathy, we discovered zinc transporters ZIP1 and ZnT1 as modifiers using a previously established *Drosophila* tauopathy model (Wittmann et al., 2001). Subsequent experiments revealed zinc affects Tau by two different means. It can bind Tau directly, affecting Tau’s properties and behaviors in a way that contributes to Tau toxicity. Also, through a mechanism distinct from binding, zinc is also involved in increased Tau phosphorylation. This latter effect appears to make a less important contribution to Tau toxicity. We conclude that, in addition to hyperphosphorylation, which has already been linked to tauopathies, direct zinc binding is another critical factor in Tau toxicity.

## RESULTS

### Zinc Reduction through Genetic or Dietary Measures Can Partially Rescue *Drosophila* Tauopathy

**Figure 1:**
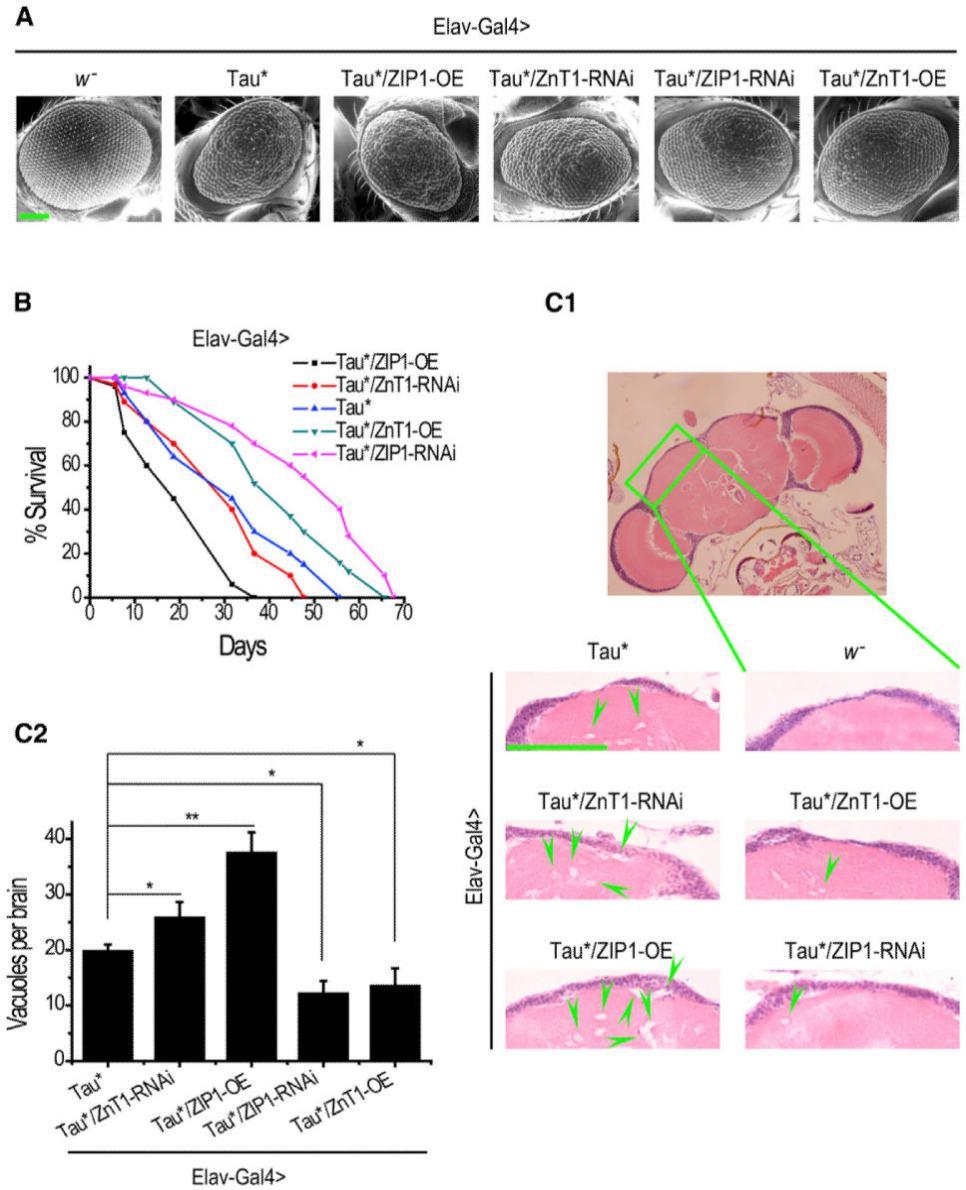
Genetic Alteration of Zinc Transporter Expression Significantly Modulates the Phenotypes of Tau Flies (A) SEM photos of Tau* flies showing the modulating effect of zinc transporters on the eyes. *Elav-Gal4* was used to drive Tau* expression in both CNS and eyes. The scale bar represents 100 μm. OE, overexpression. (B) Modulating effect of zinc transporters on the lifespans of Tau* flies. *Elav-Gal4* was used to express Tau* in the flies’ CNS. Log rank test: Tau*/ZIP1-OE versus Tau*, p < 0.0001; Tau*/ZIP1-RNAi versus Tau*, p < 0.0001; Tau*/ZnT1-OE versus Tau*, p < 0.0001; Tau*/ZnT1-RNAi versus Tau*, p < 0.01. (C1 and C2) Hematoxylin and eosin (H&E)-stained paraffin brain sections of these Tau* flies. Green arrows indicate only some of the many degenerative vacuoles formed in the brain. The scale bar represents 50 μm. (C2) is the quantification of (C1). Data represent mean ± SEM; *p < 0.05; **p < 0.01.

To investigate possible connections between metal genes and tauopathy, we set up a genetic screen to examine their interactions via a *Drosophila* tauopathy model using the bipartite upstream activating sequence (UAS)/Gal4 system. This fly tauopathy model uses hTauR406W (hereafter Tau* for short), a mutant Tau found in some FTDP-17 patients (Reed et al., 1997; Saito et al., 2002; van Swieten et al., 1999), and displays increased toxicity over wild-type Tau (Wittmann et al., 2001). A collection of overexpression or RNAi lines of genes likely relevant to metal homeostasis (Cu, Zn, and Fe) (Table S1) were analyzed. *Elav-Gal4* was used to drive the expression in the CNS. Change of eye roughness and lifespan were used as selection criteria in the screening. Several zinc transporter genes, but not genes related to other metals, were found to present consistent rescuing or enhancing effects, suggesting they act as the modifiers of *Drosophila* tauopathy. Notably, overexpression of dZnT1, a membrane zinc exporter (Wang et al., 2009), or inhibition of dZIP1, a membrane zinc importer (Lang et al., 2012), could partially suppress the rough-eye phenotype of Tau* flies (Figures 1A, Tau*/ZnT1-OE and Tau*/ZIP1-RNAi, S1A, and S1B), whereas the eye phenotype was slightly enhanced by overexpression of ZIP1 or inhibition of ZnT1 (Figures 1A, Tau*/ZIP1-OE and Tau*/ZnT1-RNAi, S1A, and S1B). In the absence of Tau*, these genetic perturbations alone did not produce noticeable eye phenotypes (Figure S1C) or obvious survival disadvantage (Figure S1D). Similar effects of the zinc transporters on Tau* toxicity were also observed when Tau* expression was directed in the eye using *Gmr-Gal4* (Figure S1E). A quantification of head transition metal levels by inductively coupled plasma-mass spectrometry (ICP-MS) revealed zinc homeostasis was indeed specifically perturbed by these genetic interventions (Figure S1F), and RT-PCR reactions were performed to confirm the efficiencies of these genetic manipulations (Figure S1G).

Considerable improvements were observed in the lifespan and brain degeneration. Overexpressing ZnT1 or knocking down ZIP1 significantly elongated the lifespan of Tau* flies, whereas overexpressing ZIP1 and, to a much-lesser extent, knocking down ZnT1 shortened the lifespan (Figure 1B). Tau* expression in *Drosophila* CNS causes vacuolization, reflecting neuronal loss and degeneration. ZIP1 overexpression or ZnT1 RNAi exacerbated the vacuolization level, whereas ZnT1 overexpression or ZIP1 knockdown significantly reduced the number of brain vacuoles (Figures 1C1 and 1C2). Therefore, genetically perturbing zinc transporters could significantly affect the neurodegenerative phenotypes in fly brains.

Having established that genetic modification of zinc transporters could suppress the defects of Tau* flies, we explored the possibility of tauopathy treatment by regulating dietary zinc uptake. Dietary zinc levels were controlled by the addition of ZnCl_2_ or CQ, a hydrophobic metal chelator (Adlard et al., 2008; Li et al., 2010), to the fly food. Metal (Cu, Fe, Mn, and Zn) content in these fly brains was measured by ICP-MS to confirm that the dietary regulation really produced the anticipated metal level changes (Figures S2A and S2B). Of note, the zinc-fed flies showed marginally reduced iron levels, implying that there could be a coregulation or interaction mechanism between zinc and iron (Solomons, 1986). CQ also slightly increased copper content, but, as previously reported, most of the copper is apparently unavailable for use so that, even when total cellular copper is increased, a state of copper deficiency is still created by CQ (Li et al., 2010).

Similar to what we observed as above in the genetic perturbation experiments, zinc addition significantly enhanced the Tau* toxicity (Figures S2C1 and S2C2), shortening the lifespan of Tau* flies (Figure S3A), whereas CQ partially rescued Tau* toxicity in *Drosophila* eyes (Figures S2C1 and S2C2) and elongated the lifespan (Figure S3B). As a control, wild-type flies (*Elav-Gal4* > *w^−^*) that were treated with the same levels of metals showed no obvious lifespan changes (Figure S3C). The neurodegeneration process of Tau* flies was also accelerated by zinc in the brain and eyes and partially mitigated by CQ (Figures S2D, S3D1, and S3D2). A quantification of the degenerative vacuoles formed per brain is shown in Figure S3D2.

Similar zinc effects were also observed in hypophosphorylated Tau mutants such as TauR406W/S262A/S356A (Tau*S2A) and TauR406W/S202A (Tau*S202A; Nishimura et al., 2004; Figures S3E and S3F). Whereas these hypophosphorylated Tau* mutants have greatly reduced toxicity and longer lifespans, their lifespans could still be significantly shortened by zinc and rescued by CQ in the case of Tau*S202A flies (Tau*S202A has stronger toxicity than Tau*S2A but less than Tau*). These results from genetic and dietary rescue studies indicate that changes in zinc homeostasis can have a partial but significant influence on the overall progression of tauopathy in vivo.

### Zinc Increases Tau Phosphorylation Level

**Figure 2:**
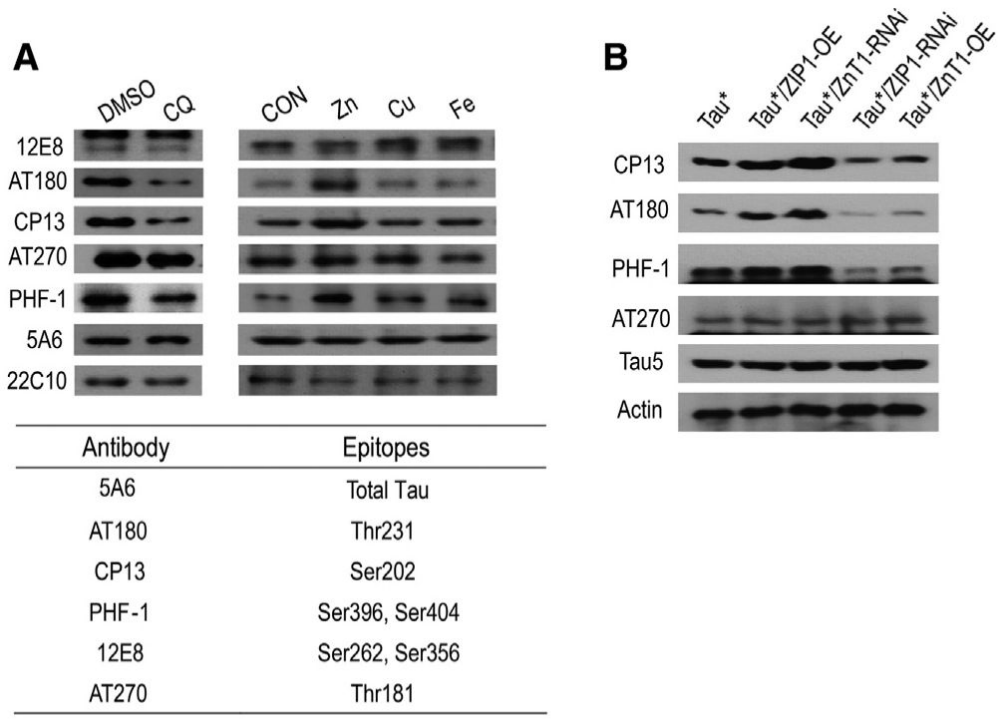
Zinc Limitation Decreases Tau Phosphorylation (A) Tau phosphorylation under different metal or CQ treatments. Antibodies 12E8, AT180, CP13, AT270, and PHF-1, as listed in the table, were used to detect different phospho-epitopes of Tau*. 5A6 was used to indicate the total Tau protein level, and 22C10 was used as an additional loading control. (B) Genetic modulation of zinc transporter genes similarly alters Tau phosphorylation. Phosphorylation levels of different epitopes of Tau* protein were detected by western blots. Results are reproducible in three independent western blotting experiments, and only one is shown here.

We next investigated how zinc could affect Tau toxicity. In AD and other tauopathies, accumulation of abnormally phosphorylated Tau is a hallmark of these diseases and is involved in fibril formation and neuron loss (Alonso et al., 1996, 2004; Hernández and Avila, 2007; Necula and Kuret, 2004). Hyperphosphorylations at serine and proline sites or threonine and proline sites have been found in disease samples (Yen et al., 1995), and several studies have suggested possible connections between zinc and Tau hyperphosphorylation (Boom et al., 2009; Sun et al., 2012). We thus analyzed Tau phosphorylation with a set of phospho-specific antibodies such as AT180, CP13, PHF-1, 12E8, and AT270, which could recognize different Tau phospho-epitopes of Tau (Dias-Santagata et al., 2007; Nishimura et al., 2004; Wittmann et al., 2001) (Figure 2A, table). Western blot results show that the AT180, CP13, and PHF-1 signals were reduced after CQ treatment and increased after zinc treatment (Figure 2A, image), three phosphorylation sites that have been previously demonstrated as important contributors to Tau toxicity.

When we genetically perturbed zinc homeostasis in the brain, similar changes of phosphorylation levels were also observed at these three sites (Figure 2B). Specifically, zinc importer ZIP1 RNAi or zinc exporter ZnT1 overexpression resulted in a reduction of phosphorylation at sites recognized by CP-13, AT180, and PHF-1 antibodies, and ZIP1 overexpression or ZnT1 RNAi gave exactly the opposite results.

We conclude that zinc could indeed affect Tau phosphorylation in the fly tauopathy model, possibly due to its effects on Tau phosphorylation pathways (Boom et al., 2009; Sun et al., 2012). But whether this phosphorylation alteration solely explains the effect of zinc on Tau toxicity still remained unanswered.

### Zinc Directly Induces Tau Protein Aggregation In Vitro

**Figure 3:**
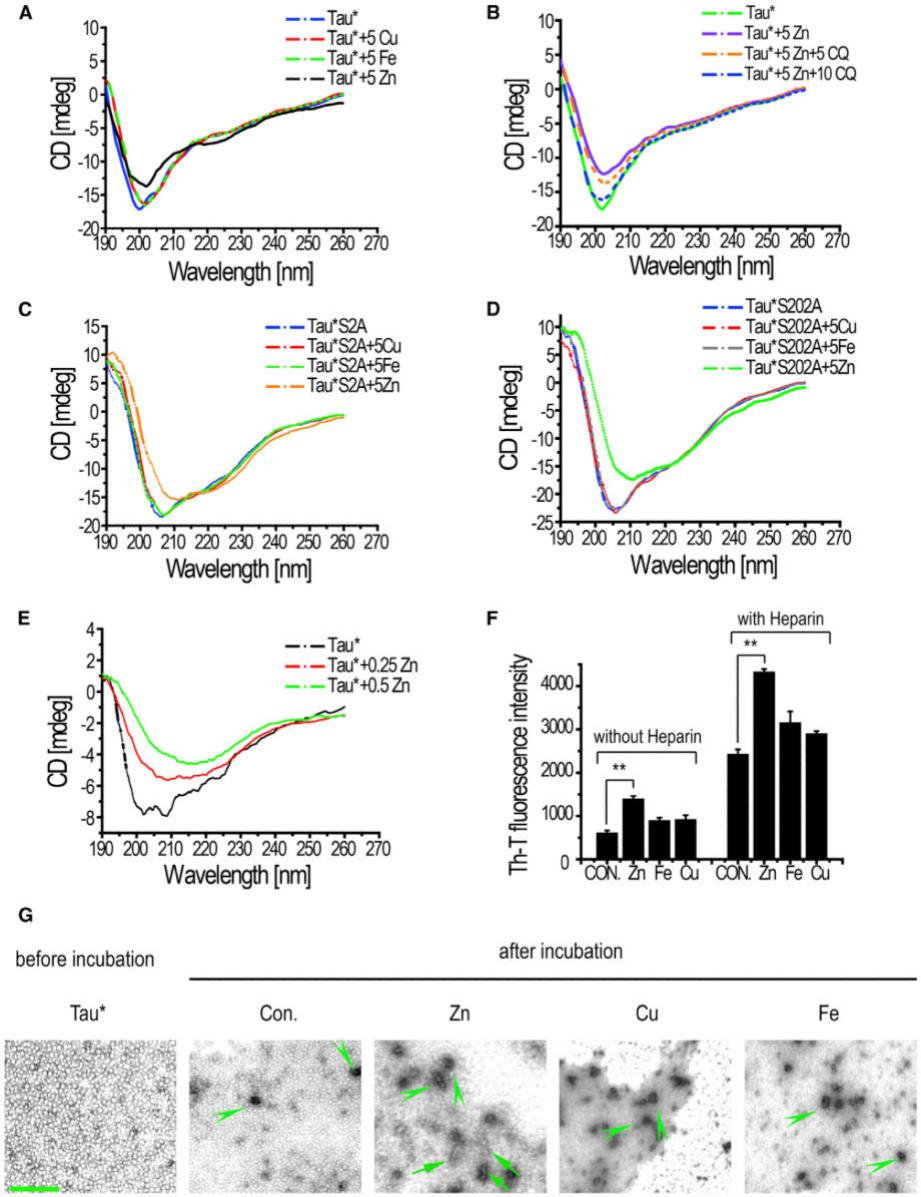
Zinc Interacts with Tau and Induces Tau Conformation Change and Aggregation In Vitro (A) Circular dichroism (CD) spectra of Tau* proteins coincubated with different metals. 5 μM copper (Cu), 5 μM iron (Fe), and 5 μM zinc (Zn) were used. Protein concentration: 70 μg/ml. (B) CD spectra of Tau* proteins incubated with Zn and CQ. 5 μM Zn was used to induce conformation change, and 5 or 10 mM CQ was used to reverse the zinc effects. Protein concentration: 70 μg/ml. (C and D) CD spectra of Tau*S202A and Tau*S2A incubated with different metals at the “low concentration” (5 μM). Protein concentration: Tau*S2A: 70 μg/ml; Tau*S202A: 90 mg/ml. (E) CD spectra of Tau* proteins under even lower Zn concentrations. Note lower concentrations of Tau* protein (40 μg/ml) and zinc (0.25 μM) were used. The reaction systems were dialyzed to remove zinc and concentrated by polyoxyethylene before measuring their CD spectra. (F) Th-T fluorescence was used to detect Tau polymerization. Tau* proteins were incubated with 5 μM Zn, 5 μM Cu, and 5 μM Fe, respectively, in the presence or absence of 1 μmol/ml heparin as a polymerization inducer. Shown here are fluorescence signal changes after 7 days of incubation. Data represent mean ± SEM. **p < 0.01. (G) TEM images of Tau* proteins coincubated with different metals. Arrowheads indicate possible Tau oligomers, and big arrows indicate small filaments formed by Tau aggregations. Protein concentration: Tau*: 70 μg/ml. The scale bar represents 200 nm.

Because zinc can bind to Tau in vitro via two Cys residues (C291 and C322; Mo et al., 2009), we wondered if zinc binding played a role in tauopathy. We used circular dichroism (CD) spectroscopy to test whether this binding could induce a secondary structure change in Tau protein. CD spectra of Tau* proteins coincubated with different metals (Cu, Fe, and Zn) did show that zinc acted as a strong promoter of Tau* conformational change (Figure 3A): the negative peak at 200 nm was reduced significantly whereas a red shift of the peak was also observed with zinc, indicating a structural change from the random coil. Although copper and iron also exhibited slight effects on Tau conformation at a higher concentration as reported previously (Ma et al., 2005, 2006), these metals failed to show any obvious effects at the lower concentration (5 μM) (Figure 3A). On the other hand, metal chelator CQ could effectively reverse the conformational change induced by zinc, returning Tau to its natural conformation (Figure 3B), as indicated by the observed changes in the 200 nm negative peak. This reversible process of CD changes suggested a potential physical binding between zinc and Tau. Conformations of hypophosphorylated Tau mutants such as Tau*S2A and Tau*S202A (Nishimura et al., 2004) could similarly be affected by zinc at the low concentration (5 μM; Figures 3C and 3D).

We next tested whether even lower zinc levels could induce Tau conformation change. Zinc concentrations as low as 0.25 or 0.5 μM, comparable to physiological zinc levels (Mocchegiani et al., 2005), could still induce significant changes in Tau* conformation (Figure 3E). The stronger promoting effect of zinc on Tau fibrillization was also confirmed by the Thioflavin T (Th-T) fluorescence assay (Figure 3F), wherein Th-T displayed an increased fluorescence when bound to aggregated Tau. The presence of heparin further enhanced the Th-T signals in general.

We additionally used transmission electron microscope (TEM) to visualize possible zinc-induced polymerizations of Tau proteins. Before incubation with metal ions, some background signals could sometimes be observed, likely resulting from small amounts of oligomers formed during protein preparation (Figure 3G). After metal incubation, Tau* proteins could be seen to form small aggregates in almost all samples (Figure 3G, green arrowheads). However, longer filament-like structures could only be found in the sample with zinc (Figure 3G, in Zn panel, green arrow). Zinc therefore, at least in vitro, could directly induce Tau* protein conformation change and promote its polymerization.

### Removal of Zinc Binding Effectively Eliminates Tau Toxicity

**Figure 4:**
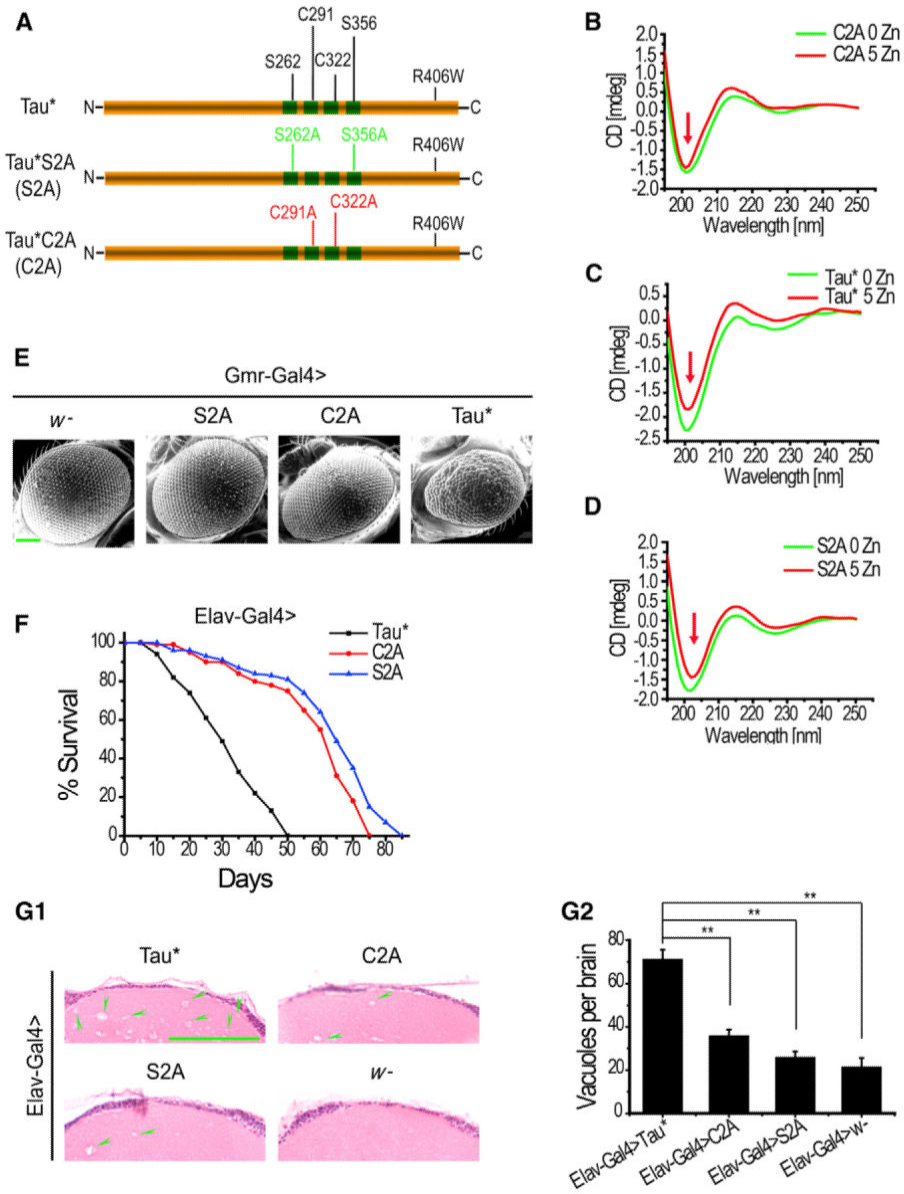
Zinc Binding Is Essential for Tau Toxicity (A) A sketch of Tau* protein showing all the relevant residues described in this work. The two zinc-binding Cys sites (Cys291 and Cys322) are represented in red, whereas the two phosphorylation sites (Ser262 and Ser356) in green. For simplicity, C2A was used to indicate Tau* protein containing Cys291Ala and Cys322Ala substitutions and S2A Tau* protein with Ser262Ala and Ser356Ala changes. (B–D) CD spectra of Tau* (protein concentration: ~8 μg/ml), Tau*C2A (protein concentration: ~8 μg/ml), and Tau*S2A (protein concentration: ~10 μg/ml) proteins coincubated with zinc. Arrows mark the approximate 200 nm peak. Apparent zinc-induced conformation changes appeared in Tau* and Tau*S2A, but not Tau*C2A. (E) Tau*C2A flies have much less affected eyes. Shown are eye scanning electron microscopy images of Tau*, Tau*S2A, and Tau*C2A flies. *Gmr-Gal4* was used to drive the eye expression of Tau. The scale bar represents 100 μm. (F) Tau*C2A flies have a much longer lifespan (comparable to that of Tau*S2A flies) than Tau*. *Elav-Gal4* was used to drive Tau* expression in CNS. The difference between Tau* and Tau*C2A or Tau*S2A lifespans was significant, p < 0.0001 (log rank test). (G1 and G2) H&E-stained paraffin brain sections of Tau*, Tau*C2A, and Tau*S2A flies. The scale bar represents 50 μm. Green arrowheads indicate some of the degenerated vacuoles. Statistical analysis of the results is shown in G2. Data represent mean ± SEM; **p < 0.05.

Because zinc is an essential nutritional element, removing it entirely from a cell or organism is unfeasible. Dramatic loss, as well as increase, of intracellular zinc inevitably results in cell death. To better demonstrate in vivo whether the direct zinc binding to Tau is functionally relevant, site-specific mutagenesis was utilized to replace potential zinc-binding sites (Mo et al., 2009) (Figure 4A, C291A and C322A, red sites) in the Tau* protein. Single mutants C291A and C322A and double mutant C291A/C322A (Tau*C2A for short) were generated and compared with Tau*S2A (Figure 4A, S262A and S356A, green sites) or Tau* itself for toxicities. C291A and C322A single-substitution mutants displayed almost identical phenotypes as Tau*C2A double mutant (Figure S4). Here, we will present only Tau*C2A as examples. As a control, we showed that the mutant Tau*C2A exhibited a similar CD profile as that of Tau* (Figure S5A), suggesting little conformation change results from these amino acid replacements. Immunostaining also revealed that Tau*C2A colocalizes with microtubules in the *Drosophila* motor neuron axon and muscle, similar to Tau* and Tau*S2A (Figures S5B1 and S5B2). Further, coimmunoprecipitation confirmed all three proteins (Tau*, Tau*C2A, and Tau*S2A) are able to bind to the microtubule components, again suggesting normal functions are retained in Tau*C2A (Figure S5C). However, in vitro CD spectra analysis indicated Tau*C2A is no longer able to bind to zinc (Figure 4B), in contrast to Tau* (Figure 4C) and Tau*S2A (Figure 4D).

Ectopically expressed Tau*C2A and Tau*S2A displayed dramatically reduced levels of toxicity in fly eyes in comparison to Tau* (Figures 4E, S5D, and S5E). In the lifespan assays, we also observed similarly prolonged survival of Tau*C2A (mean lifespan about 60 days) and Tau*S2A flies (mean lifespan about 63 days), when compared with Tau flies (mean lifespan about 30 days) (Figure 4F). In order to rule out differences in protein expression, we determined that all three chosen lines express comparable amounts of target proteins. Brain sections from Tau*, Tau*C2A, and Tau*S2A flies were additionally examined for vacuole formations. Far fewer vacuoles were found in Tau*C2A and Tau*S2A flies brains, indicating a great reduction of Tau*-induced neurodegeneration (Figures 4G1 and 4G2).

### Loss of Zinc Binding Confers Tau Inertness to Zinc Changes

**Figure 5:**
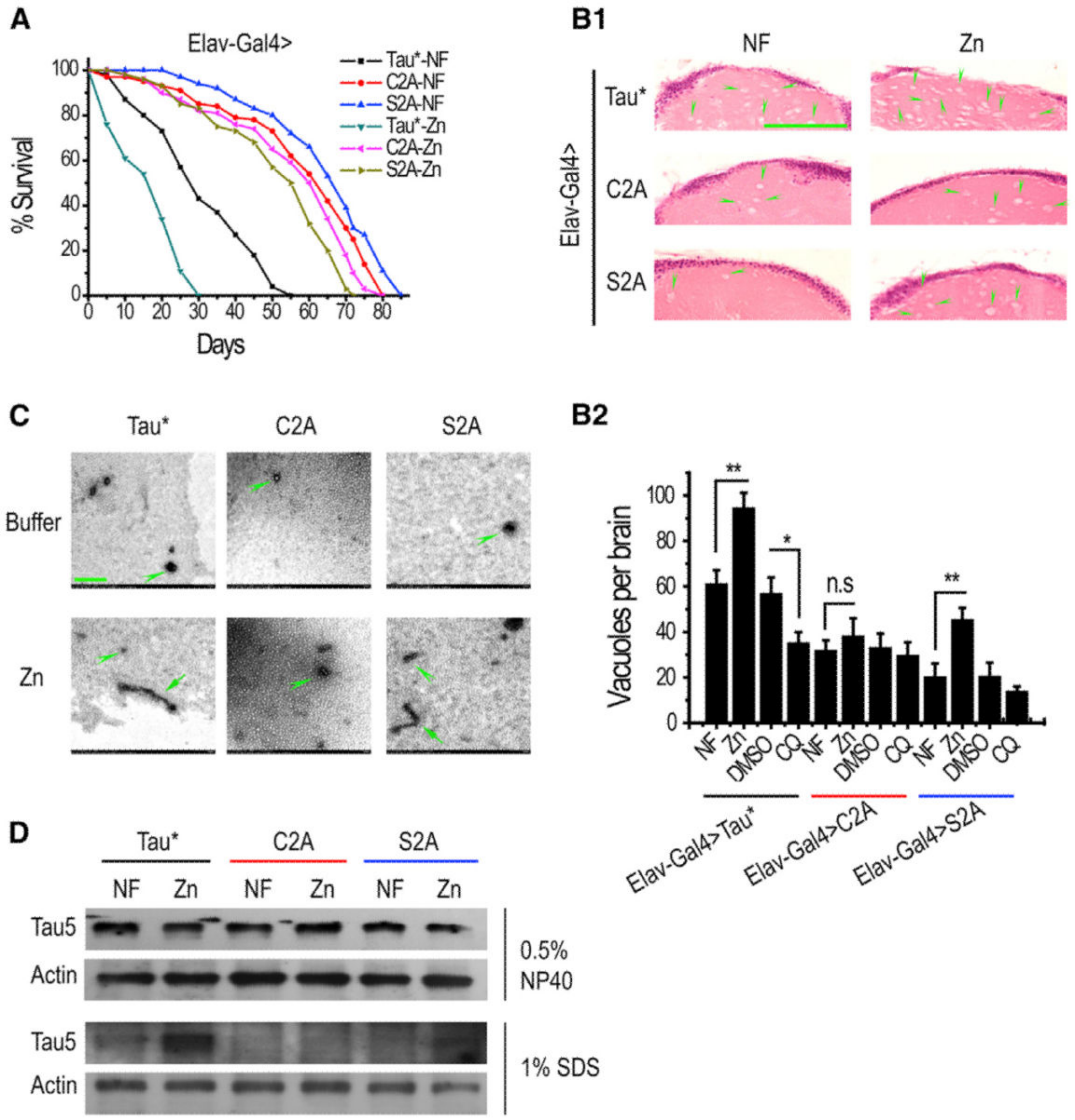
Substitution of Zinc Binding Residues Essentially Eliminates the Zinc Responsiveness of Tau Toxicity (A) Lifespans of Tau*C2A flies are essentially inert to zinc changes. Shown here are Tau*, Tau*S2A, and Tau*C2A flies under zinc treatment. *Elav-Gal4* was used to express proteins in the CNS. Tau*-NF versus Tau*-Zn, p < 0.0001; Tau*S2A-NF versus Tau*S2A-Zn, p < 0.0001; Tau*C2A-NF versus Tau*C2A-Zn, p ≈ 0.05. NF, normal food. (B1 and B2) Brain degeneration is not significantly affected by zinc treatment in Tau*C2A flies. Shown here are H&E-stained paraffin brain sections of Tau*, Tau*C2A, and Tau*S2A flies under zinc treatment. The scale bar represents 50 μm. Green arrowheads indicate some of the many degenerated vacuoles. A quantitative and statistical analysis of B1 is shown in B2. Data represent mean ± SEM and were compared with NF group within each genotype. *p < 0.05; **p < 0.01. n.s., p > 0.05, not significant. (C) Tau*C2A does not respond to zinc incubation in vitro. TEM images of Tau*, Tau*S2A, and Tau*C2A proteins coincubated with zinc are presented. The scale bar represents 200 nm. The concentrations of Tau*, Tau*S2A, and Tau*C2A were adjusted to ~50 μg/ml before the metal incubation. (D) Zinc treatment increases the insoluble species of Tau protein in vivo. Insoluble Tau fractions were extracted from fly heads using the extraction buffer containing 1% SDS and detected by Tau5 antibody. Actin was used as a control to show comparable loadings among samples.

If loss of toxicity for Tau*C2A is the result of loss of zinc binding, and for Tau*S2A from phosphorylation reduction, we expect the zinc responses of Tau*C2A, Tau*S2A, and Tau* flies to be different. Indeed, unlike that of Tau* and Tau*S2A flies, zinc could not appreciably affect the lifespan of Tau*C2A flies (Figure 5A). However, for similarly nontoxic Tau*S2A, which is able to bind zinc, zinc-responsiveness was still apparent (Figure 5A). Paraffin brain sections also revealed that zinc could not influence the toxicity of Tau*C2A: when treated with zinc, the neurodegeneration in Tau*C2A flies did not noticeably worsen as measured by the number of vacuoles formed in the brains (Figures 5B1 and 5B2).

The effect of zinc on these Tau* variants was reproduced with in vitro binding assays. When incubated with zinc, both Tau*S2A and Tau* formed aggregates whereas Tau*C2A failed to do so (Figure 5C, arrowheads for small aggregations and arrows for filament-like aggregations). Polymerization of these Tau* variants was also examined in vivo. Zinc treatment significantly increased the amount of Tau in the insoluble fraction for both Tau* and Tau*S2A (Figure 5D) but without obvious effect on Tau*C2A (Figure 5D).

Interestingly, normal *Drosophila* Tau (dTau) lacks Cys (Figure S6A). Therefore, it is likely that the zinc-binding Cys residues may not be necessary for normal Tau functions, consistent with our CD and microtubule binding results as reported above. However, it would be worthwhile to test whether the toxicity from dTau overexpression is responsive to zinc level change. Again, as we observed with Tau*C2A, dTau toxicity is largely insulated from zinc level alterations (Figure S6B).

Taken together, these results indicate loss of zinc binding effectively suppresses Tau toxicity from deleterious zinc-modulating effects.

### Restoration of Zinc Binding to Tau Correlatively Restores Its Toxicity and Zinc Responsiveness

**Figure 6:**
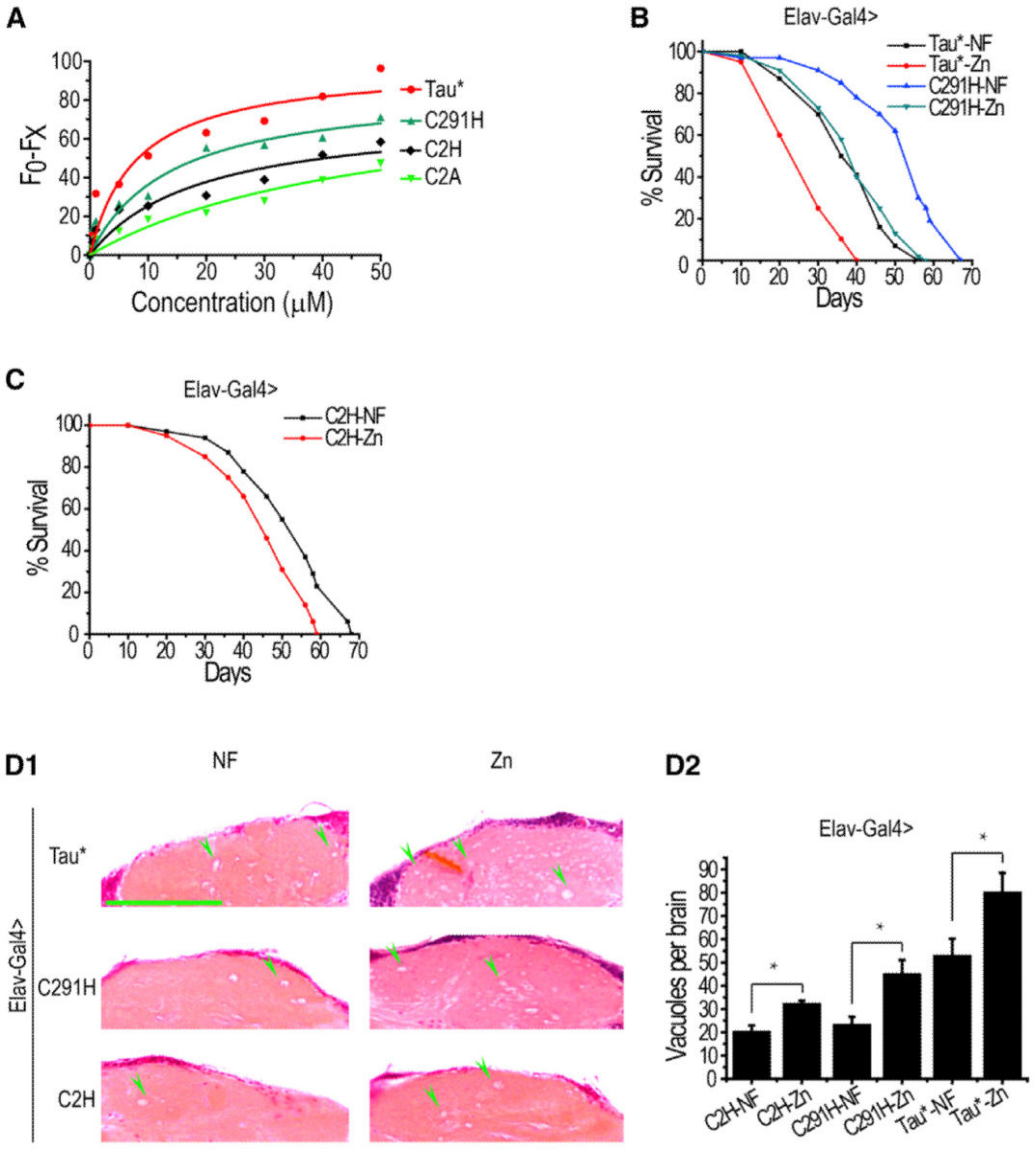
Restoration of Zinc Binding to Tau Regains Correlatively Its Zinc Responsiveness (A) Various forms of Tau* upon zinc binding as detected by intrinsic fluorescence quenching. Fluorescence excited at 280 nm was measured after 5 min equilibration. (B) Lifespan of Tau*C291H flies versus zinc. Zinc supplementation can enhance Tau*C291H toxicity to a level approaching that of Tau under normal diets. *Elav-Gal4* was used to drive Tau expression. Tau*-NF versus Tau*-Zn, p < 0.0001; Tau*C291H-NF versus Tau*C291H-Zn, p < 0.0001. (C) Lifespan of Tau*C2H flies. Comparing with that of Tau*C2A, the lifespan of Tau*C2H flies can still be affected by zinc. Tau*C2H-NF versus Tau*C2H-Zn, p < 0.01. (D1 and D2) Brain degeneration was also affected by zinc treatment in Tau*C291H and Tau*C2H flies. Shown here are H&E-stained paraffin brain sections of Tau*, Tau*C291H, and Tau*C2H flies under zinc treatment. A quantitative and statistical analysis is shown in D2. The scale bar represents 50 μm. Green arrowheads indicate only some of the many degenerated vacuoles. Data represent mean ± SEM; *p < 0.05; **p < 0.01.

One caveat associated with mutating Cys to Ala in Tau* is that, in addition to loss of zinc binding, the amino acid substitution might also affect other biological aspects of Tau. To address this question, we made additional Tau* mutants: Tau*C291H and Tau*C2H (C291H and C322H). We reasoned that, if loss of zinc binding is indeed the sole factor attributed to the decrease in toxicity, Tau*C291H (relative to Tau*C291A) or Tau*C2H (relative to Tau*C2A) may regain zinc-binding ability as histidine residues are also often utilized by proteins to bind zinc. If this were the case, we would expect these mutants to restore zinc-responsive toxicity in Tau. When assayed by fluorescence quenching, the two Tau* mutants exhibited better zinc response than the non-zinc binding Tau*C2A, though neither as well as Tau* itself. Tau*C2H, in particular, binds zinc significantly more weakly (Figure 6A). Both exhibited very little toxicity under normal conditions when assayed by longevity (Figures 6B and 6C). However, when the zinc levels were increased, Tau*C291H and to a lesser extent Tau*C2H regained toxicity (Figures 6B and 6C). In fact, in the survival assay, elevated zinc concentrations caused Tau*C291H to exhibit a level of toxicity nearly comparable to that of Tau* under nonelevated zinc concentrations. Brain sectioning revealed similar results (Figures 6D1 and 6D2). These results are in contrast with what was observed with C to A changes, which were relatively insulated from zinc alterations. The correlation between Tau* toxicity and zinc-binding ability, together with the Tau*C2A and dTau results, provide strong support for our model that the toxicity loss associated with mutating cysteine residues results from an attenuation or loss in zinc binding, but not other factors.

### Zinc Binding and Phosphorylation Are Two Independent Events for Tau

**Figure 7:**
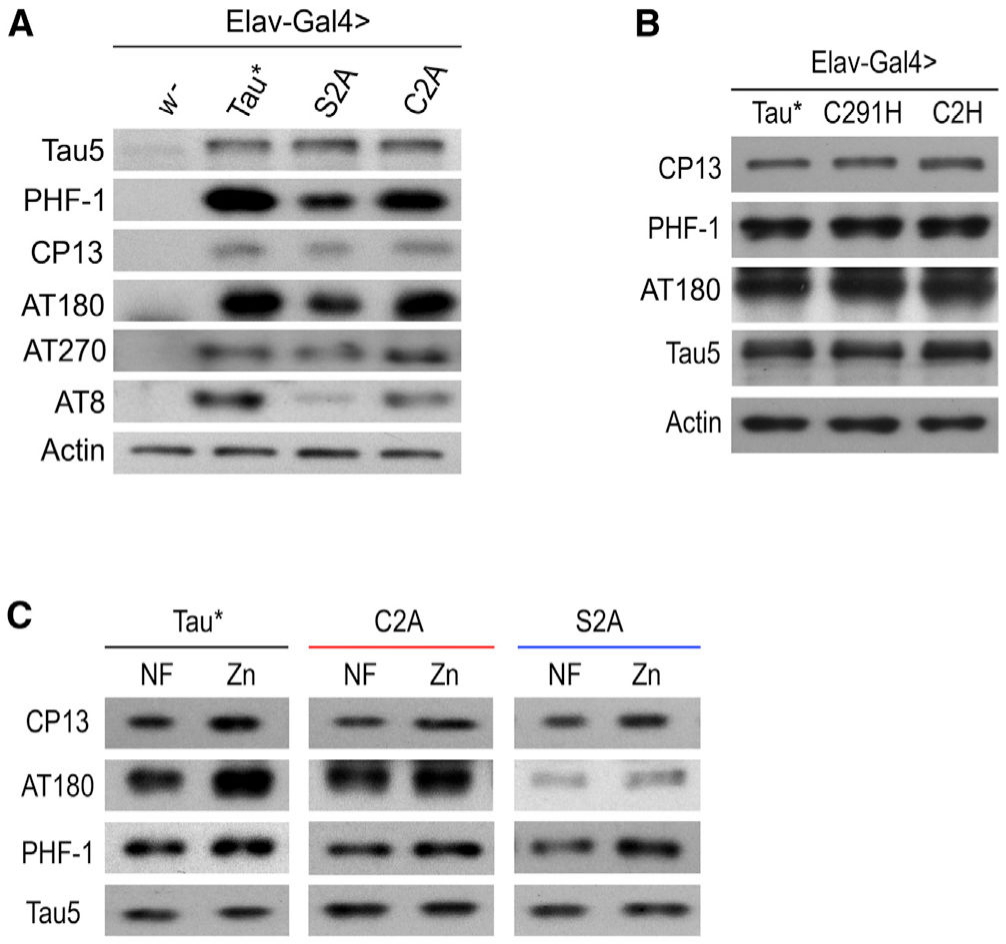
Zinc Binding to Tau and Its Effect on Tau Phosphorylation Are Two Independent Events (A) Tau*C2A undergoes similar phosphorylation as Tau*. Shown are phosphorylation levels of Tau*, Tau*S2A, and Tau*C2A proteins as detected by western blots. (B) Tau*C2H and Tau*C291H also undergo similar phosphorylation as Tau*. Shown are phosphorylation levels of Tau*, Tau*C2A, and Tau*C291H proteins. (C) Phosphorylation of Tau*C2A is similarly affected by zinc. *Elav-Gal4* was used to express the proteins in CNS. Tau5 was used to indicate the total Tau level, and actin was used as an additional loading control.

If zinc binding is essential for the manifestation of Tau toxicity, we asked if zinc binding might increase Tau phosphorylation, resulting in elevated toxicity, whereas loss of zinc binding could reduce phosphorylation. If this were the case, our zinc-binding mutant Tau*C2A would show lower levels of phosphorylation. To explore this possibility, we examined the phosphorylation levels of Tau*C2A, Tau*S2A, and Tau* proteins by immunoblotting. To our surprise, Tau*C2A maintained comparable levels of phosphorylation with that of Tau*. In fact, both Tau* and Tau*C2A showed much higher phosphorylation levels than Tau*S2A (Figure 7A), whose loss of toxicity is attributed to phosphorylation reduction (Nishimura et al., 2004). Likewise, we also observed comparable phosphorylation among Tau*, Tau*C291H, and Tau*C2H (Figure 7B). These results indicate that the reduced toxicity observed in Tau*C2A is not due to Tau phosphorylation changes; instead, it is caused by the abolished zinc binding per se.

As previously demonstrated, Tau*C2A toxicity is relatively insulated from changes in zinc concentration. However, when we probed the phosphorylation levels of Tau*C2A in vivo, we found that, similar to Tau*, it still displayed the zinc-induced increase in phosphorylation at several phospho-epitopes (Figure 7C), confirming that zinc binding to Tau and zinc’s enhancing effect on Tau phosphorylation are indeed two distinct and separable actions. Phosphorylation in Tau*S2A is also affected by zinc exposure (Figure 7C), indicating Tau phosphorylation is at least partially independent of PAR-1, because phosphorylation of S2A is not attributed to PAR-1 (Nishimura et al., 2004). Consistently, previous findings indicate zinc regulates Tau phosphorylation indirectly, likely through affecting several kinase pathways, like Erk (Kim et al., 2011), p70S6K (An et al., 2005), and phosphatase PP2A (Sun et al., 2012; Xiong et al., 2013). In summary, our results have uncoupled zinc binding and zinc-induced phosphorylation as two independent factors in Tau toxicity.

## DISCUSSION

Zinc contributes to Tau’s toxicity by two independent ways. First, zinc indirectly affects Tau phosphorylation likely through the action of kinase or phosphatase pathways. In another, more important aspect, it directly binds to Tau and serves a critical role for Tau toxicity. By mutating zinc-binding residues of Tau, we were able to distinguish between zinc’s two modes of action. Amazingly, removal of zinc binding almost completely abolishes Tau toxicity, suggesting that zinc binding and hyperphosphorylation equally contribute to tauopathy. We propose that appreciable Tau toxicity requires both the presence of hyperphosphorylation and zinc binding (Figure S7).

Previously, zinc’s effect on Tau was assumed to be auxiliary (Boom et al., 2009; Budimir, 2011; Greenough et al., 2013). The lack of in vivo evidence and the indispensability of zinc for normal cellular functions made evaluating zinc’s role in tauopathy a challenge. Through our set of carefully designed experiments, we were able to clearly separate the phosphorylation effect and zinc-binding effect and establish zinc’s key role in the pathogenesis of tauopathy. The effect of zinc binding is otherwise hard to discern due to the concomitant effect of zinc on Tau phosphorylation and because zinc modulation, by genetic or chemical measures, must be limited to avoid causing detrimental effects in vivo.

Although our results show that zinc has an effect on Tau hyperphosphorylation pathways, this effect appears to be less important to Tau toxicity than zinc binding directly to Tau. This conclusion is clearly supported by results from the reduced toxicity of the Tau*C2A mutant, which cannot bind zinc but exhibits normal zinc-responsive phosphorylation. Despite zinc-responsive phosphorylation changes, Tau*C2A phenotypes are not much affected by zinc level alterations. In comparison, Tau*S2A, which shows reduced levels of phosphorylation but maintains its ability to bind zinc, shows greater toxicity in response to increases in zinc.

Previous in vitro studies show that the two Cys residues altered in Tau*C2A may be also involved in intramolecular and intermolecular Cys-Cys crosslinking actions. Specifically, substitution of C291A suppresses intracellular disulfide bond formation and leads to elevated intermolecular crosslinking and toxicity, whereas C322A diminishes toxicity (Schweers et al., 1995). According to the crosslinking model, C291A should be more toxic than even the normal Tau. However, in our hands, both C291A and C322A presented little toxicity in vivo, suggesting the change of crosslinking cannot explain the loss of toxicity here. To further show that it is indeed zinc binding that contributes to Tau toxicity, we further made Tau*C291H and Tau*C2H mutants. These two mutants should not have the proposed crosslinking ability as conferred by Cys residues in Tau*; however, they displayed zinc-responsive toxicity as Tau*, in accordance with their zinc-binding abilities.

Several metals (copper, iron, and zinc) possess the ability to bind Tau peptides and induce aggregations in vitro (Boom et al., 2009; Ma et al., 2005, 2006; Mo et al., 2009; Yamamoto et al., 2002), yet no genetic evidence has ever been given to substantiate the functional relevance of these interactions in vivo. In this study, we used a *Drosophila* model to verify these interactions between metals and tauopathy. Only zinc among the three transition metals listed showed a toxicity-enhancing effect in vivo. It is possible that the other metals bind Tau with less affinity and the internal cellular environment does not provide high enough levels of these ions.

Religa et al. (2006) reported that elevated cortical zinc is a feature of advanced AD, correlating with increased cognitive deterioration and highest plaque burden. We found Aβ expression itself is sufficient to induce zinc accumulation in the fly head (Lang et al., 2012), whereas little overall increase was observed in tauopathy flies (data not shown). Therefore, in AD, a combination of Aβ plaque and tauopathy, with the increased zinc level as a result of Aβ accumulation, could accelerate late Tau toxicity development, leading to advanced AD.

The apparent zinc involvement in both tauopathy and Aβ-generated toxicity (Cherny et al., 2001; Lang et al., 2012; Suh et al., 2000) has led researchers to question whether zinc reduction would be even more effective in AD treatment. Indeed, chelating therapy at the organismal level has shown some promising signs in AD clinical trials (Budimir, 2011; Duce and Bush, 2010). Our use of CQ also significantly improved the pathological findings of Tau flies. However, because zinc is such an important cofactor ubiquitously involved in many fundamental biological reactions, reduction of zinc to a critical level may substantially affect the corresponding processes and become deleterious to organism’s survival. Indeed, interfering with the expression of some of the zinc transporters can even lead to the death of the organism. Thus, although zinc reduction is effective in mitigating Tau toxicity, zinc’s full role in tauopathy may not be fully uncovered due to limited physiologically allowable zinc reduction. In other words, the reduction of Tau toxicity is significantly less dramatic through zinc reduction than zinc binding removal, suggesting Tau’s zinc binding is with relatively high affinity and zinc-Tau binding is still prevalent even under reduced zinc conditions. Compared to the organism-wide zinc reduction through dietary uptake limitation, more stringent restriction of zinc in only pathologically affected regions is worth consideration. In addition, a much more effective therapy would involve specifically perturbing the interaction between Tau and zinc, for example, with a small molecule inhibitor that binds to Tau’s zinc-binding region. Our current study is an important step forward toward understanding the etiology of tauopathy and may aid in the future design of more-effective treatment strategies.

## EXPERIMENTAL PROCEDURES

### *Drosophila* Stocks and Genetics

General fly stocks used in this study were obtained from the Bloomington *Drosophila* Stock Center. *UAS-TauR406W* (UAS-Tau*), *UAS-TauR406W* (*S202A*) (UAS-Tau*S202A), and *UAS-TauR406W*(*S2A*) (UAS-Tau*S2A) flies were as previously described (Nishimura et al., 2004). For all the physiological and biochemical assays, *Gmr-Gal4* flies were crossed with UAS-Tau* flies to ectopically express Tau* in the eyes, and *Elav-Gal4* flies were crossed with UAS-Tau* flies to express Tau* in the CNS. Flies were raised in standard corn meal food under 25(±1)° C unless noted.

### Chemical Treatment and Lifespan Recording

For lifespan recordings, newly eclosed adults were raised on standard corn media with or without metal or other chemical supplements. In metal treatment experiments, a final concentration of 1 mM ZnCl_2_, 1 mM FAC (ammonium ferric citrate), or 0.25 μM CuCl_2_ was added to the diet; in CQ treatment, flies were fed with 0.5 μM CQ diluted from a stock solution dissolved in DMSO, and in this case, 1% DMSO was used as the corresponding control. Lifespans were recorded at 25° C. At least 100 flies were used for each individual experiment.

### Site-Directed Mutagenesis and Fly Transformation

pUAST vector containing human Tau(R406W) (Tau*) was used as the template to generate various Tau mutants via site-directed mutagenesis. Constructs were sequence confirmed and transformed into fly *w^1118^*(*w*^−^) background. Multiple transgenic lines were obtained and inserts confirmed by genomic PCR. Protein expression levels were determined by western blot with Tau5 antibodies (mouse 1:1,500; Invitrogen).

### Immunoblotting

To analyze phosphorylation levels of Tau proteins, adult fly heads were homogenized in the lysis buffer as described (Nishimura et al., 2004). Protein extracts were separated by 12% SDS-PAGE. The samples on polyvinylidene fluoride membranes were then incubated with antibodies at the following dilutions: 5A6 (mouse 1:2,000), PHF-1 (mouse 1:1,500), CP13 (rabbit 1:2,000), AT270 (mouse 1:1,500), AT180 (rabbit 1:1,500), 12E8 (mouse 1:2,500), 22C10 (mouse 1:500), Tau5 (mouse 1:1,500), and actin (mouse 1:1,500). PHF-1 and 5A6 were from the Hybridoma Bank (University of Iowa), and the other antibodies were derived as described (Nishimura et al., 2004) or purchased from Invitrogen. Secondary antibodies were peroxidase-labeled antimouse immunoglobulin G (IgG), or anti-rabbit IgG, and signals were developed by enhanced chemiluminescence (Pierce). Nonionic detergent soluble and insoluble Tau fractions were prepared following a previously described method (Khurana et al., 2010). Equivalent amounts of soluble and insoluble proteins from different groups were loaded for the western blotting and probed by Tau5 (mouse 1:1,500) and actin (mouse 1:1,500) antibodies.

### Protein Expression, Purification, and Circular Dichroism Spectra Measurement

Human Tau(R406W) 0N4R form of Tau*, Tau*C2A, Tau*C2H, Tau*C291H, and Tau*S2A coding regions were cloned into pMXB10 (New England Biolabs) vector. Proteins were purified by chitin beads (NEB) as described (Venter et al., 2011; Wang et al., 2010) and concentrations measured by the BCA kit (Thermo Scientific). In the CD assay, proteins were incubated with zinc or other metals at 37° C in 0.01 M PBS (pH = 7.4) buffer containing 1 mM dithiothreitol (DTT). CD spectra were measured in the far ultraviolet region (190–260 nm) and recorded by the Jasco J-715 Spectropolarimeter.

### Th-T-Binding Assay

To analyze metal-induced Tau polymerization, freshly prepared 0.6 μmol Tau* protein (about 24 μg) was incubated with or without metals (5 μM Cu, Fe, or Zn) in 200 μl 20 mM Tris-HCl buffer containing 1 mM DTT (pH = 7.4) for 1 week. Th-T (Sigma-Aldrich) solution was freshly prepared, filtered, and then added to a final 20 μM concentration. Fluorescence of Th-T was recorded at 440 nm excitation and 480 nm emission by the luminescence spectrometer (SYNERGY4; Gene Company; Mo et al., 2009).

### Fluorescence Quenching Assay

We assayed 0.05 μg purified protein in 500 μl buffer (0.01 M PBS; 1 mM DTT [pH = 7.4]) for changes in fluorescence intensity. Zinc was added stepwise, and emission readings were collected. Fluorescence was monitored at ~348 nm following excitation at 280 nm using a F4500 Fluorescence Spectrometer (Zundel et al., 1998).

### Histology

For paraffin sections, fly heads were fixed in Carnoy fixation solution (ethanol: chloroform:acetic acid = 6:3:1) for 4 hr at the room temperature, then dehydrated sequentially by 100% ethanol for 30 min twice, dry ethanol (100% ethanol dried with desiccant) and methyl benzoate each for 1 hr, and stepwise embedded in melted paraffin. The paraffin-embedded fly heads were sectioned into 8 μm continuous sections. Hematoxylin-eosin (ZSGB-BIO) staining was used to facilitate the observation of the vacuoles in the brain samples.

### TEM and Scanning Electron Microscopy

Freshly prepared Tau proteins were incubated overnight with or without metals at 37° C in 0.01 M PBS buffer containing 1 mM DTT (pH = 7.4). Uranyl-acetate-negative staining was performed to facilitate subsequent TEM observations. Aggregate formation was examined by transmission electron microscope (H-7650B). Scanning electron microscopy analysis of fly eyes was performed as described previously (Chatterjee et al., 2009) with FEI Quanta 200.

### Metal Content Analysis

Sixty adult heads were collected, and metal contents were assayed using the ICP-MS XII (Thermo Electron) as described previously (Tang and Zhou, 2013). Data were normalized with weights of the samples.

### Statistics

Data are presented as mean ± SEM. Differences among groups were analyzed by the IBM SPSS v13.0 with Student’s t (comparison of two groups) or ANOVA test (three groups or more). *p < 0.05; **p < 0.01. Differences between lifespans were analyzed by the log rank method using the GraphPad Prism 5 software, *p < 0.05; **p < 0.01; ***p < 0.0001; not significant (n.s.): p > 0.05.

## Supplementary Material

Cell_report_suppl
