# Supplementary material for: Zinc Binding Directly Regulates Tau Toxicity Independent of Tau Hyperphosphorylation
Source: Cell Rep. Author manuscript; Available in PMC 2015 Jan 26. (PMC4306234; doi:10.1016/j.celrep.2014.06.047)
Supplement: Cell_report_suppl [file NIHMS656169-supplement-Cell_report_suppl.pdf]

Cell Reports, Volume *8*

Supplemental Information

Zinc Binding Directly Regulates Tau Toxicity Independent of Tau Hyperphosphorylation

Yunpeng Huang, Zhihao Wu, Yu Cao, Minling Lang, Bingwei Lu, and Bing Zhou

Supplementary information

Figure S1. Expression Modulation of Zinc Transporters Affects Tau Phenotypes.

(Related to Figure 1)

(A) Semi-quantification of rough eye phenotype, related to the SEM pictures shown in Figure 1A. *Elav-Gal4* was used to drive Tau* expression in fly eyes, and 100 progenies were observed of each genotype. -, +, ++ and +++ mean normal, weak, medium and strong rough eye phenotype respectively. (B) Tangential sections of fly compound eyes. *Elav-Gal4* was used to drive Tau* expression in the eyes. Scale bar: 20μm. (C) SEM photos of fly eyes when zinc transporters alone were overexpressed or RNA interfered. No obvious difference was seen when compared with the control w^- flies. Scale bar: 100μm. (D) The effect of zinc transporters on fly lifespans when modulated alone. No difference from the control w^- flies was observed (p>0.05, Log-rank test). (E) SEM photos of Tau* flies when eye driver *Gmr-Gal4* was used. Scale bar: 100μm. (F) Brain metal contents of Tau* flies when zinc transporters were genetically interfered with. Data represent mean±SEM; *: p<0.05. (G) Efficiencies of OE or RNAi analyzed by RT-PCR. *Elav*-Gal4 was used to drive Tau* and zinc transporter expression or knocking down.

Figure S2. Dietary Zinc Limitation Mitigates Tau Eye Phenotype.

(Related to Figure 1)

(A, B) Brain metal contents of Tau* flies under metal and CQ treatments. Data represent mean±SEM, *: p<0.05, **: p<0.01. (C1) SEM photos of Tau* flies

with different metal or chelator treatments. *Gmr-Gal4* was used to express Tau* in the eyes. Final concentrations of different metals used: 1mM $ZnCl_2$, 1mM FAC or 0.25 mM $CuCl_2$; clioquinol (CQ, a metal chelator) treatment: 0.5 mM CQ. NF (normal food) and DMSO (food with 1% DMSO) were used as controls for metal and CQ treatment respectively. (C2) A quantification of Supplementary Figure S2C1. (D) Tangential sections of fly compound eyes. *Gmr-Gal4* was used to drive Tau* expression in the eye. Scale bar: 20μm.

Figure S3. Dietary Zinc Limitation Increases Tau Fly Lifespan.

(Related to Figure 1)

(A, B) Lifespans of Tau* flies with metal or chelator treatment. *Elav-Gal4* was used to express Tau* in the central nervous system (CNS). NF vs Zn, $p<0.0001$; NF vs Cu or Fe, $p>0.05$; DMSO vs CQ, $p<0.0001$. (C) Control flies (*Elav-Gal4>w-*) with the same metal or chelator treatment, $p>0.05$. (D1, D2) H&E stained paraffin brain sections of Tau* flies with different zinc treatments. Green arrowheads indicate some of the many vacuoles formed from degenerated neurons. Scale bar: 20μm. D2 is the quantification of D1. Data represent mean±SEM, *: $p<0.05$. (E, F) Lifespans of Tau*S2A and Tau*S202A flies with metal or chelator treatment. These hypophosphorylated mutant Tau* flies are still responsive to zinc modulation. Tau*S2A-NF vs Tau*S2A-Zn, $p<0.0001$; Tau*S202A-NF vs Tau*S202A-Zn, $p<0.0001$; Tau*S202A-NF vs Tau*S202A-CQ, $p<0.0001$.

Figure S4. The Lifespans of Tau*C291A and Tau*C322A Flies.

(Related to Figure 4)

Elav-Gal4 was used to express the various forms of Tau in the CNS. The lifespans of Tau*C291A and Tau*C322A flies were both significantly improved over that of the Tau*. Tau*C332A vs Tau*, $p<0.0001$; Tau*C291A vs Tau*, $p<0.001$.

Figure S5. Tau*C2A Retains Similar Conformation and Function of Tau*.

(Related to Figure 4)

(A) Circular dichroism (CD) spectra of Tau* and Tau*C2A proteins. The conformation of Tau*C2A is minimally changed in comparison to that of Tau*. (B1, B2) Tau*C2A, Tau*S2A and Tau* exhibited similar cellular localizations. Tau*C2A, Tau*S2A and Tau* localizations were compared in the *Drosophila* motor neuron axon (B1) and muscle (B2). *Elav-Gal4* was used to express Tau in the neuron and *C57-Gal4* in the muscle; all were co-stained with the microtubules. Scale bars: 25μm (B1) and 40μm (B2). (C) Tau*C2A, Tau*S2A and Tau* could all bind to the microtubule component (α-Tubulin) *in vivo.* Tau*, Tau*C2A and Tau*S2A proteins were pulled down with human Tau antibody. Anti-human Tau used in the immunoprecipitation was from Pierce, and all other antibodies used in the western blots (1:1000) were from Santa Cruz Biotechnology. (D) A quantification of Figure 4E. *Gmr-Gal4* was used to drive Tau expression in the eyes. (E) Tangential sections of flies' compound eyes. Both Tau*C2A and Tau*S2A mutants have little eye abnormalities. *Gmr-Gal4* was used to drive Tau* expression in the eyes. Scale bar: 20μm.

Figure S6. Zinc's Effect on dTau Flies' Lifespan.

(Related to Figure 5)

(A) Sequence comparison between dTau and hTau showing dTau carries no corresponding Cys residues. Sequence alignment was performed using the Clustal W, and the corresponding Cys (red arrow head) region was shown here. dTau: *Drosophila* Tau; hTau: human Tau. (B) dTau flies are also relatively resistant to the zinc's effect. *Elav-Gal4* was used to express dTau in the CNS. The lifespans of dTau, Tau* and w^- were recorded on NF/Zn food. Tau*: hTauR406W; dTau: *Drosophila* Tau. Tau*-NF vs Tau*-Zn, $p<0.0001$; dTau-NF vs dTau-Zn, $p>0.05$.

Figure S7. A Model of Tau Toxicity and Zinc's Role in Tauopathy.

(Related to Figure7)

(A) Phosphorylation and zinc binding are both essential for Tauopathy. Zinc binding to Tau is an independent event from the phosphorylation process. Lacking either of the two critical factors could effectively diminish Tau toxicity. (B) Dual effects of zinc on Tau toxicity. In addition to its binding to Tau, zinc also indirectly affects Tau phosphorylation. While zinc can affect Tau phosphorylation indirectly, this effect on phosphorylation may not be the major effect zinc has on Tau toxicity, since without zinc binding (Tau*C2A) Tau toxicity is greatly reduced and no longer appreciably enhanced by zinc despite increased phosphorylation. The fact that Tau*C291H manifests a significant toxicity only when zinc is increased corroborates this viewpoint.

Figure S1

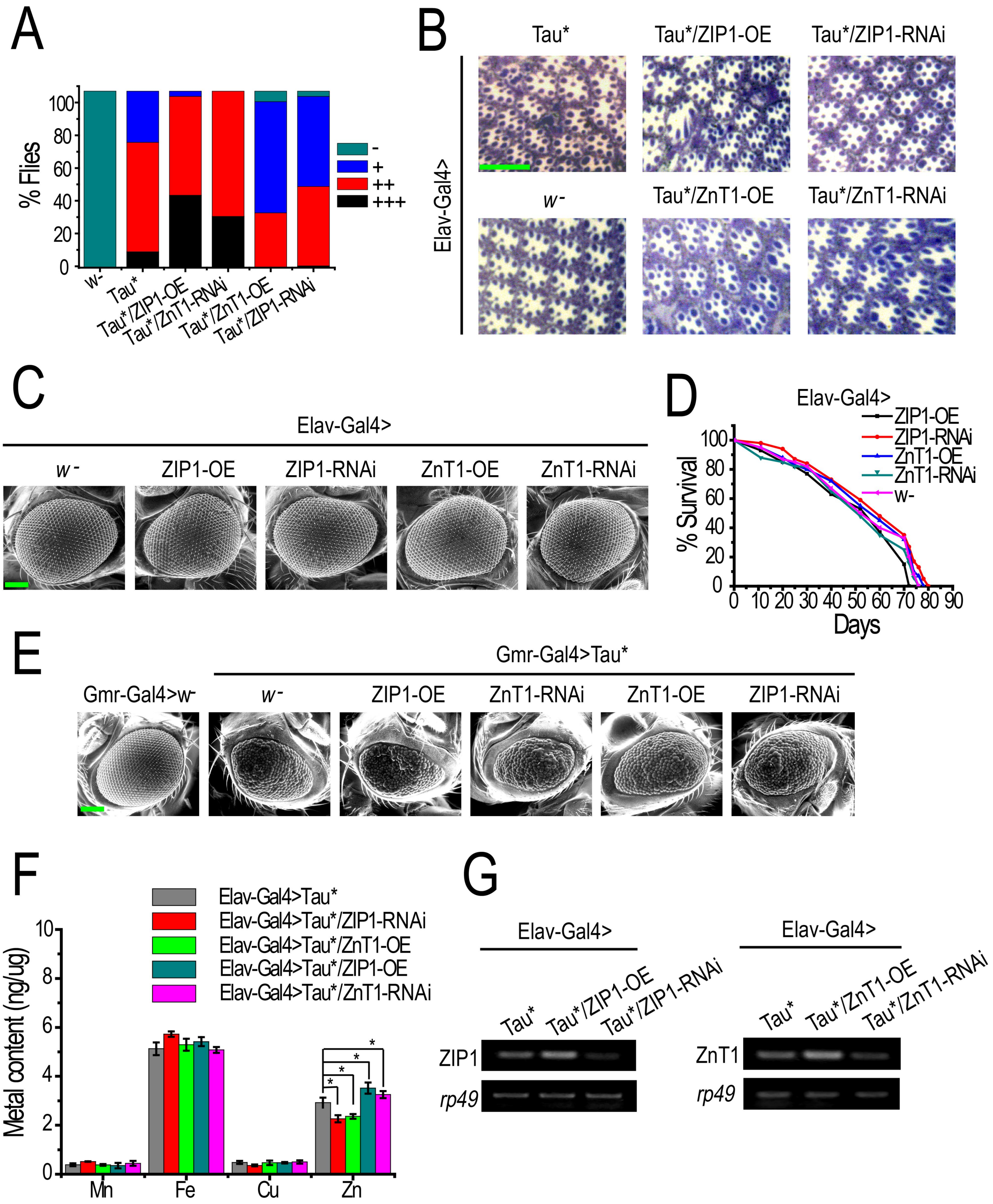
A
% Flies
-
+
++
+++
w-
Tau*
Tau*/ZIP1-OE
Tau*/ZnT1-RNAi
Tau*/ZnT1-OE
Tau*/ZIP1-RNAi
B
Tau*
Tau*/ZIP1-OE
Tau*/ZIP1-RNAi
Elav-Gal4>
w-
Tau*/ZnT1-OE
Tau*/ZnT1-RNAi
C
Elav-Gal4>
w-
ZIP1-OE
ZIP1-RNAi
ZnT1-OE
ZnT1-RNAi
D
Elav-Gal4>
ZIP1-OE
ZIP1-RNAi
ZnT1-OE
ZnT1-RNAi
w-
% Survival
Days
E
Gmr-Gal4>Tau*
Gmr-Gal4>w-
w-
ZIP1-OE
ZnT1-RNAi
ZnT1-OE
ZIP1-RNAi
F
Elav-Gal4>Tau*
Elav-Gal4>Tau*/ZIP1-RNAi
Elav-Gal4>Tau*/ZnT1-OE
Elav-Gal4>Tau*/ZIP1-OE
Elav-Gal4>Tau*/ZnT1-RNAi
Metal content (ng/ug)
Mn
Fe
Cu
Zn
G
Elav-Gal4>
Tau*
Tau*/ZIP1-OE
Tau*/ZIP1-RNAi
ZIP1
rp49
Elav-Gal4>
Tau*
Tau*/ZnT1-OE
Tau*/ZnT1-RNAi
ZnT1
rp49

Figure S2

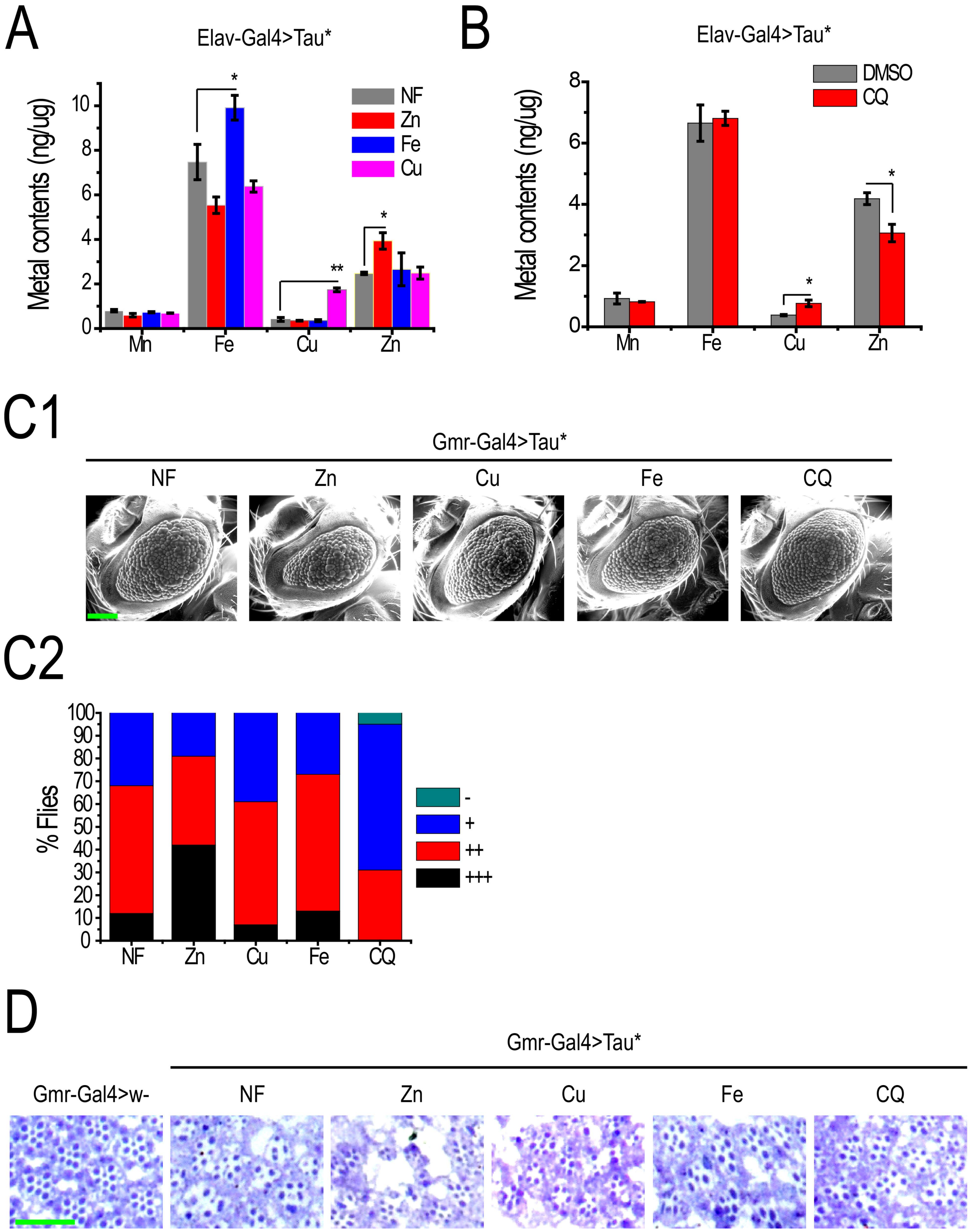
A
Elav-Gal4>Tau*
Metal contents (ng/ug)
NF
Zn
Fe
Cu
Mn
B
DMSO
CQ
C1
Gmr-Gal4>Tau*
C2
% Flies
-
+
++
+++
D
Gmr-Gal4>w-

Figure S3

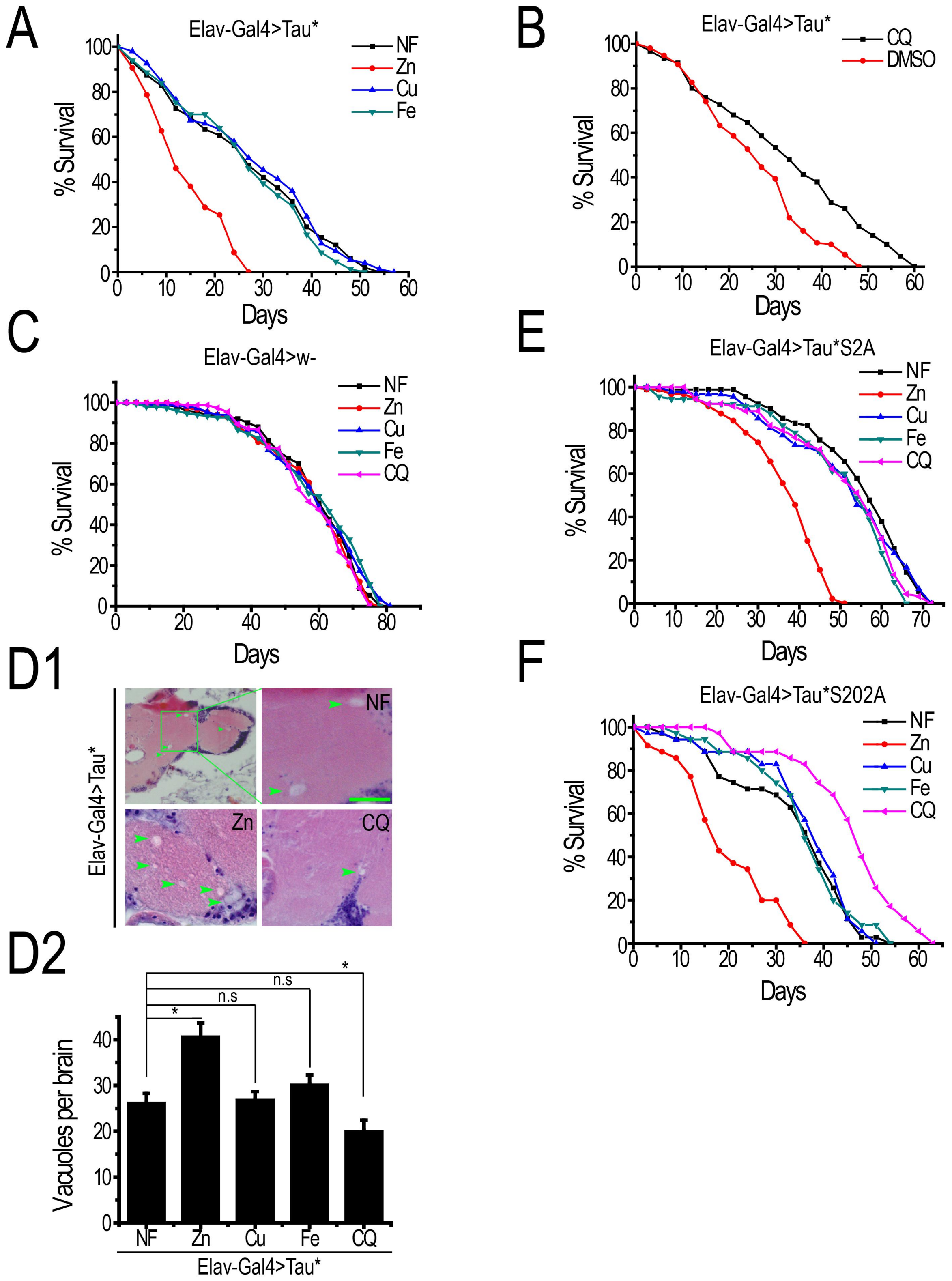
A
Elav-Gal4>Tau*
NF
Zn
Cu
Fe
% Survival
Days
B
Elav-Gal4>Tau*
CQ
DMSO
% Survival
Days
C
Elav-Gal4>w-
NF
Zn
Cu
Fe
CQ
% Survival
Days
E
Elav-Gal4>Tau*S2A
NF
Zn
Cu
Fe
CQ
% Survival
Days
D1
Elav-Gal4>Tau*
NF
Zn
CQ
F
Elav-Gal4>Tau*S202A
NF
Zn
Cu
Fe
CQ
% Survival
Days
D2
Vacuoles per brain
*
n.s
n.s
*
NF
Zn
Cu
Fe
CQ
Elav-Gal4>Tau*

Figure S4

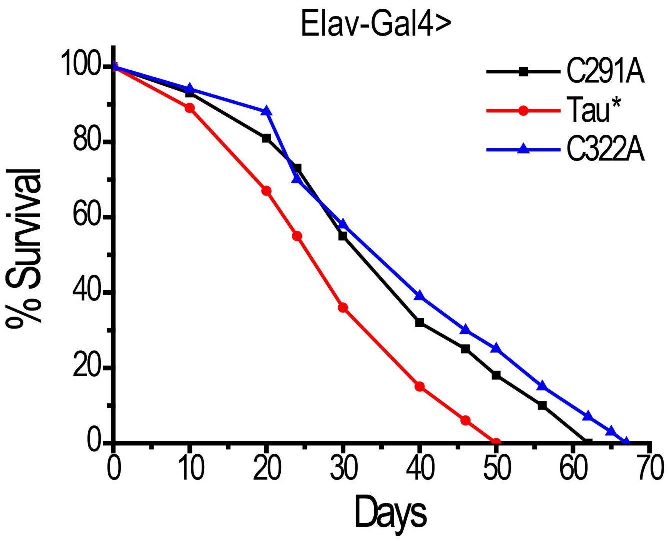
Elav-Gal4>
C291A
Tau*
C322A
% Survival
Days
0
20
40
60
80
100
0
10
20
30
40
50
60
70

Figure S5

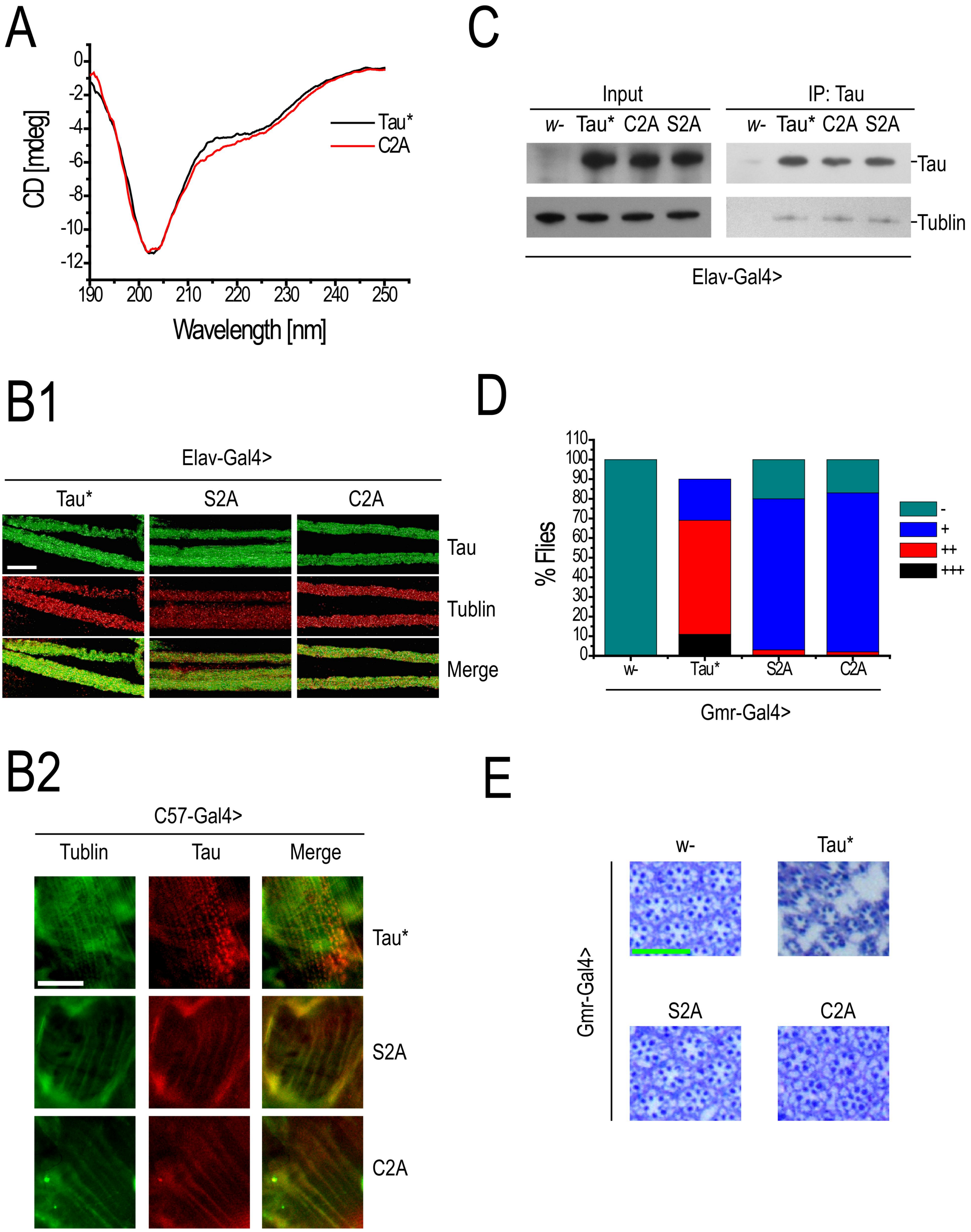
A
0
-2
-4
-6
-8
-10
-12
CD [mdeg]
190
200
210
220
230
240
250
Wavelength [nm]
Tau*
C2A
C
Input
IP: Tau
w- Tau* C2A S2A
w- Tau* C2A S2A
Tau
Tublin
Elav-Gal4>
B1
Elav-Gal4>
Tau*
S2A
C2A
Tau
Tublin
Merge
D
110
100
90
80
70
60
50
40
30
20
10
0
% Flies
w-
Tau*
S2A
C2A
Gmr-Gal4>
-
+
++
+++
B2
C57-Gal4>
Tublin
Tau
Merge
Tau*
S2A
C2A
E
w-
Tau*
S2A
C2A
Gmr-Gal4>

Figure S6

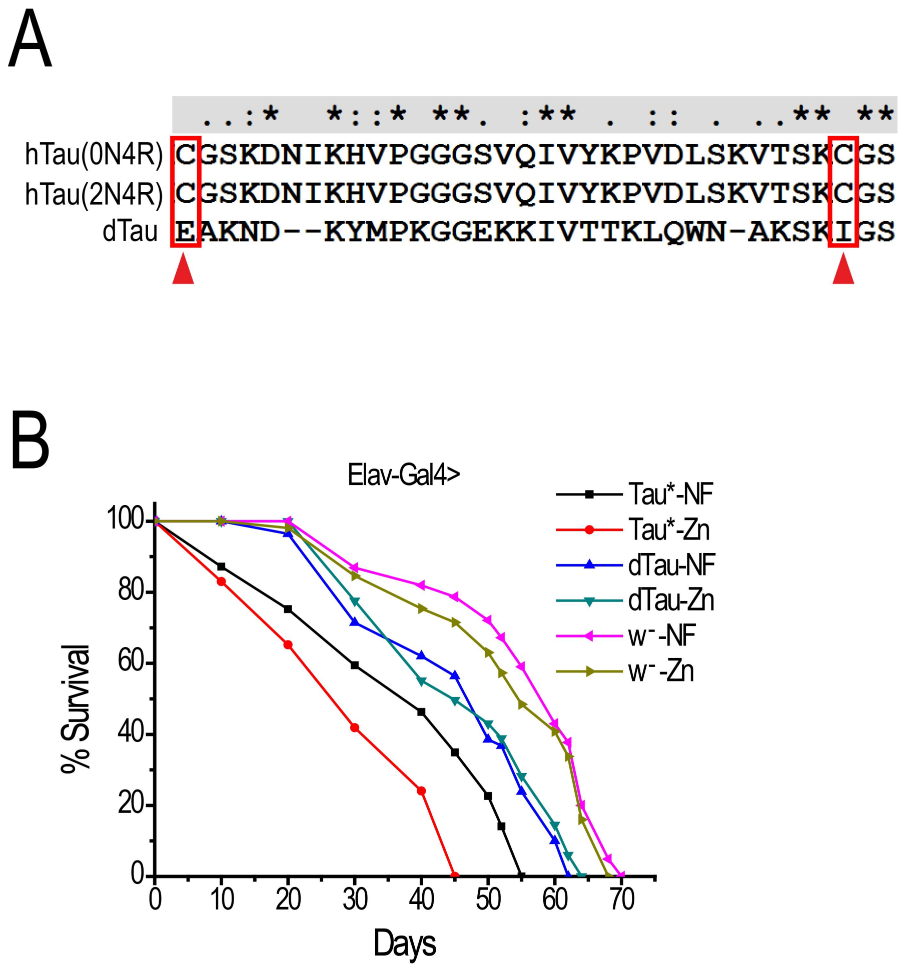
A
hTau(0N4R) CGSKDNIKHVPGGGSVQIVYKPVDLSKVTSKCGS
hTau(2N4R) CGSKDNIKHVPGGGSVQIVYKPVDLSKVTSKCGS
dTau EAKND--KYMPKGGEKKIVTTKLQWN-AKSKIGS
B
Elav-Gal4>
Tau*-NF
Tau*-Zn
dTau-NF
dTau-Zn
w⁻-NF
w⁻-Zn
% Survival
Days
0
20
40
60
80
100
0
10
20
30
40
50
60
70

Figure S7

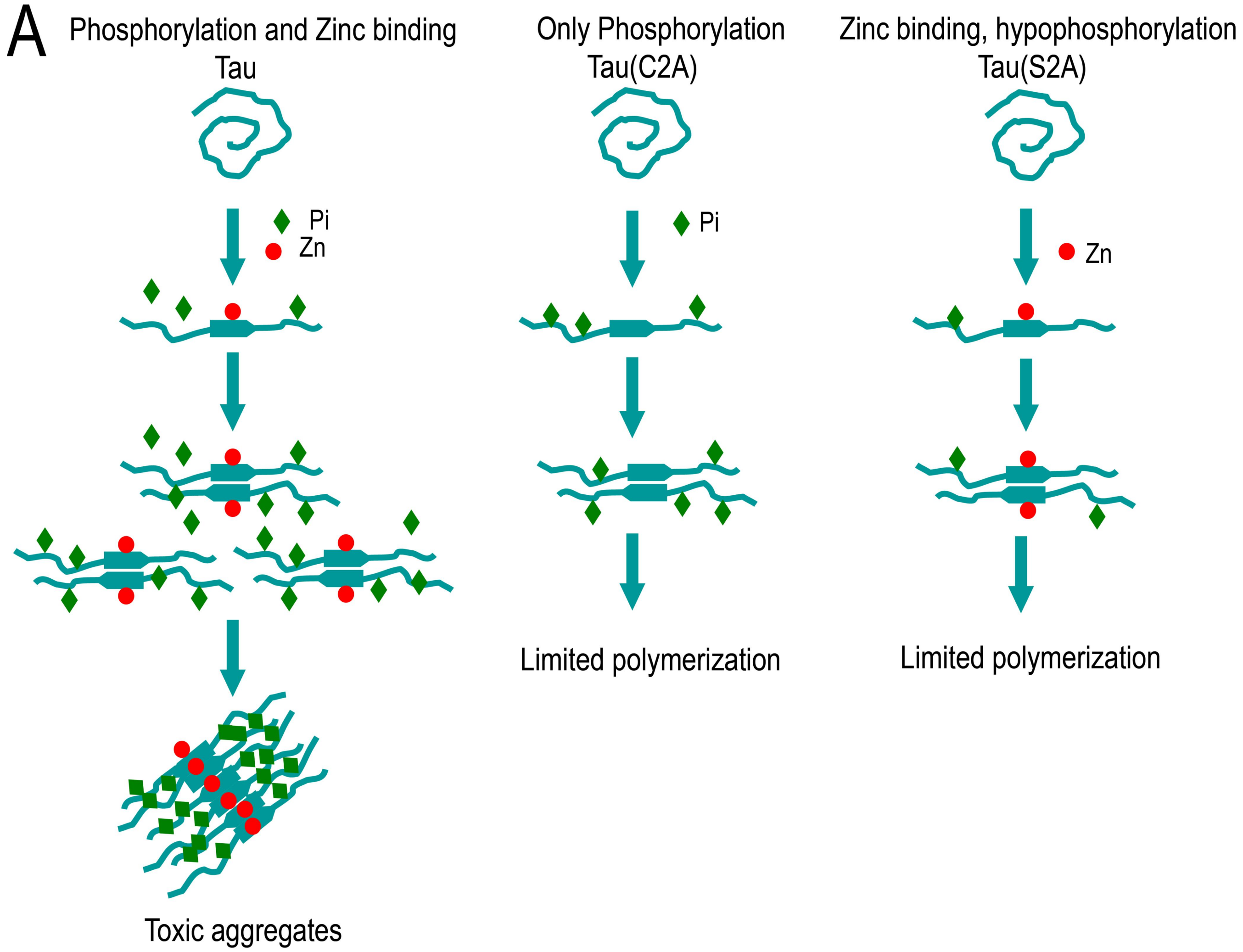
A
Phosphorylation and Zinc binding
Tau
Pi
Zn
Toxic aggregates
Only Phosphorylation
Tau(C2A)
Pi
Limited polymerization
Zinc binding, hypophosphorylation
Tau(S2A)
Zn
Limited polymerization

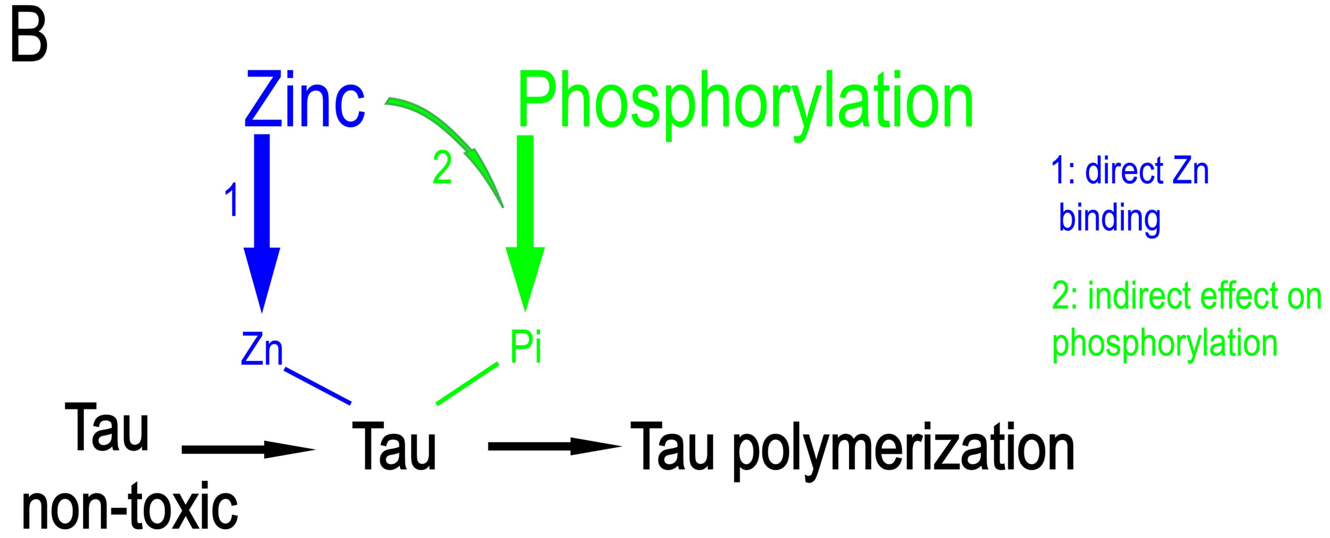
B
Zinc
Phosphorylation
1
2
Zn
Pi
1: direct Zn binding
2: indirect effect on phosphorylation
Tau non-toxic
Tau
Tau polymerization

Table S1. Metal Homeostasis Genes Used for Tau Modifier Screening

(Related to Figure 1)

	Ctr1A (CG3977)		*ZIP1 (CG9428)*<br>*ZIP2 (CG9430)*		*Fer1HCH (CG2216)*<br>*Fer2LCH (CG1469)*
	Ctr1B (CG7459)		*ZIP3 (CG6898)*		*Fer3HCH (CG4963)*
	Ctr1C (CG15551)		*ZnT1 (CG17723)*		*Malvolio (CG3671)*
	ATP7 (CG1886)		*CG4334 (homolog of hZIP3)*		*Tsf1 (CG6186)*
	ATX1 (CG32446)		*foi (CG6817)*		*Tsf2 (CG10620)*
	CCS1 (CG17753)		*CG10006 (homolog of hZIP5)*		*frataxin homolog (CG8971)*
**Cu**	*COX17 (CG9065)*	**Zn**	*CG7816 (homolog of hZIP13)*	**Fe**	*CG32557 (homolog of yeast FET3)*
	SCO1 (CG8885)		*Catsup (CG10449, homolog of hZIP7)*		*MCO3 (CG5959, homolog of yeast FET3)*
			CG11163 (homolog of hZnT2)		*laccase2 (CG42345, homolog of yeast FET5)*
	COX11 (CG31648)		*CG5130 (homolog of hZnT1)*		*ferrochelatase (CG2098)*
